# Age Estimation of African Lions *Panthera leo* by Ratio of Tooth Areas

**DOI:** 10.1371/journal.pone.0153648

**Published:** 2016-04-18

**Authors:** Paula A. White, Dennis Ikanda, Luigi Ferrante, Philippe Chardonnet, Pascal Mesochina, Roberto Cameriere

**Affiliations:** 1 Center for Tropical Research, Institute of the Environment and Sustainability, University of California, Los Angeles, California, United States of America; 2 Tanzania Wildlife Research Institute, Arusha, Tanzania; 3 Center of Epidemiology, Biostatistics and Medical Information Technology, Department of Biomedical Science and Public Health, Polytechnic University of Marche, Ancona, Italy; 4 IGF-International Foundation for Wildlife Management, Paris, France; 5 AgEstimation Project, Institute of Legal Medicine, University of Macerata, Macerata, Italy; University of Southern Queensland, AUSTRALIA

## Abstract

Improved age estimation of African lions *Panthera leo* is needed to address a number of pressing conservation issues. Here we present a formula for estimating lion age to within six months of known age based on measuring the extent of pulp closure from X-rays, or Ratio Of tooth AReas (ROAR). Derived from measurements taken from lions aged 3–13 years for which exact ages were known, the formula explains 92% of the total variance. The method of calculating the pulp/tooth area ratio, which has been used extensively in forensic science, is novel in the study of lion aging. As a quantifiable measure, ROAR offers improved lion age estimates for population modeling and investigations of age-related mortality, and may assist national and international wildlife authorities in judging compliance with regulatory measures involving age.

## Introduction

The African lion *Panthera leo* is a species for which there is growing need for improved age assessments. Recent concerns over lion population declines have prompted increased interest in population dynamics, and in particular, causes of mortality [1–4]. Accurate age estimates are essential to demographic studies [5, 6] because age data contribute to understanding population dynamics [7], serve to inform age-specific life-tables [8, 9], and are needed to refine models used to monitor population trends [1]. Improved age estimation of lions can also help in determining the potential impacts that different sources of mortality may impart at the population level. For example, determining the ages of “problem” lions killed as a result of human-wildlife conflicts [10, 11], and the ages of lions represented in the growing trade in lion bones [12], may improve our understanding of the subsequent impacts of these losses on lion populations.

Age is also a key factor in debates over trophy lion hunting. While hunting can play a vital role in conservation [13, 14], unsustainable offtake has been implicated in localized population declines [3, 15, 16]. Age-based trophy selection, whereby it is required that hunted animals meet or exceed a minimum age standard, is considered an essential component of sustainable harvest of African lions [17, 18]. However, while increasingly countries that allow hunting of lions are adopting very specific minimum age restrictions that are legally binding [19, 20], the techniques currently available for assessing age offer assignment of adults to age class only [21, 22]. International regulatory authorities i.e., USFWS [4] and EU-SRG [23] also have vested interest in ensuring that their citizens comply with foreign laws and regulations, including trophy age standards e.g., Lacey Act [24]. Thus, improved age estimation of lions is needed to better monitor population trends, investigate age-related mortality, guide sustainable management practices and assist national and international wildlife authorities with regulatory enforcement.

Dentition provides reliable cues for age estimation in mammals [25–27]. In African lions, eruption of permanent dentition is usually completed by two years of age, with the exception of the canines that are not fully erupted until about three years of age [21]. Closure of the apical foramina in lion canine teeth occurs between 28–36 months of age; lower canines close before the upper canines, and closure occurs a few months earlier in females than in males [21].

There is evidence that closure of the pulp chamber in lions increases with age in canine teeth [21] and in the second upper premolar (PM^2^)[28, 29]. However, while X-rays of PM^2^s are routinely consulted when assessing lion age [22, 30], to our knowledge, the relationship between pulp closure in PM^2^s and known age has not been quantified for the species. It is also unclear whether complete pulp chamber ossification occurs in lion PM^2^s, or should reasonably be expected to occur in all individuals. Tooth cementum annuli line counts have proven accurate for age determination in many species [31]. However, recently it was shown that cementum line counts in PM^2^s were unreliable for lions [29].

The aim of this study was to develop a formula for age estimation using Cameriere’s method [32] to measure the pulp/tooth area ratio of the PM^2^ of known-aged lions. The resultant Ratio Of tooth AReas (ROAR) formula would be applicable to PM^2^ measures of lions for whom ages are unknown for purposes of improved age estimation. This formula is anticipated to be broadly useful for studies of lion populations. Additionally, it provides wildlife management authorities with a more quantifiable means by which ages of individual lion trophies can be assessed to ensure compliance with age restrictions, thereby promoting conservation of wild lion populations.

## Results

### Repeatability of Measures

**Fig 1:**
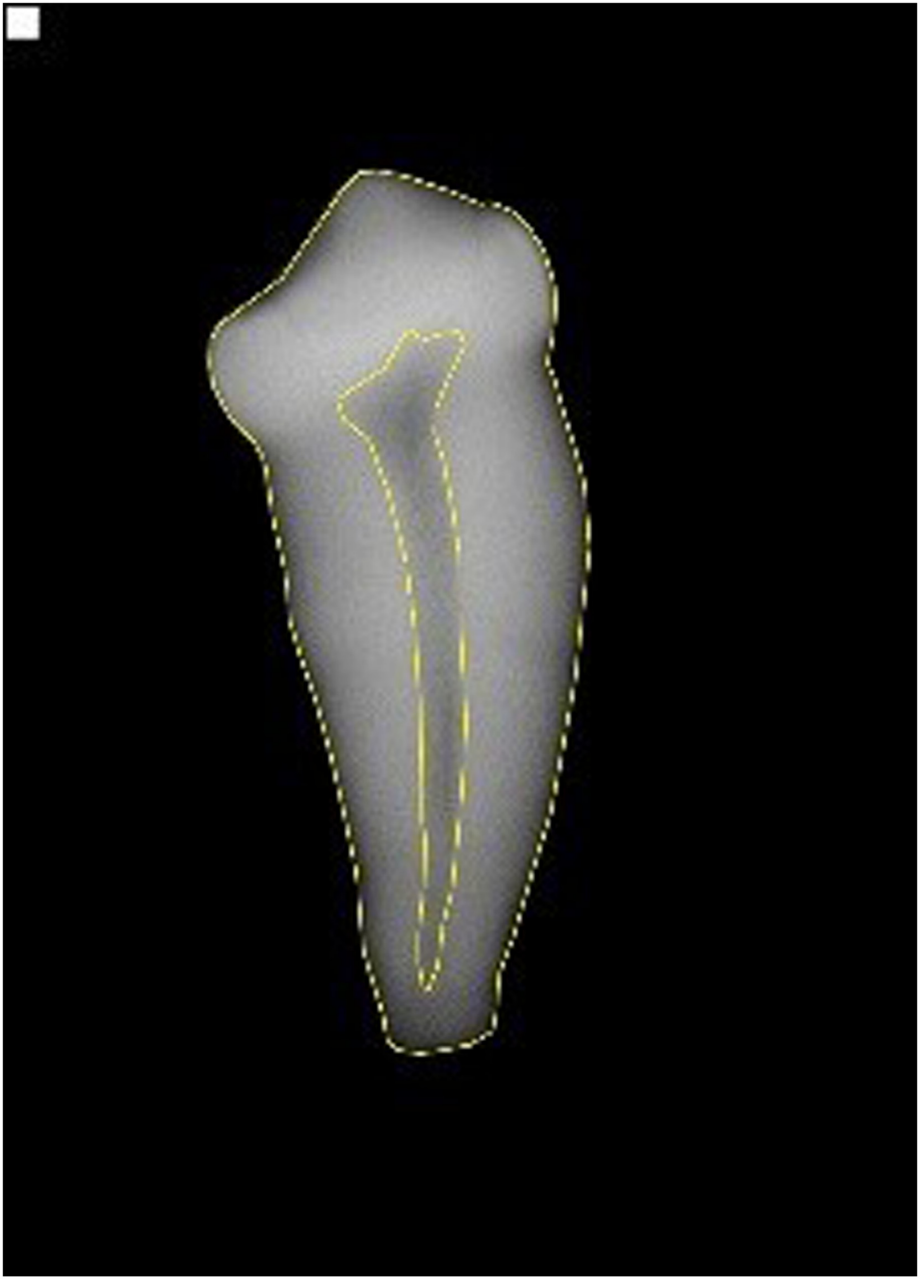
Peri-apical X-ray of African Lion PM^2^. X-ray showing outline of total tooth and pulp areas for calculating area ratio using Adobe Photoshop after Cameriere et al. (2011) [33]. Full details of the outlining and area measuring method are provided in S1 Text.

An intra-class correlation coefficient (ICC) of 0.962 and its non-parametric bootstrapped 95% confidence interval (95% CI: 0.902–0.988) indicated an acceptable level of intra-observer agreement between paired sets of measurements carried out on re-examined X-rays (Fig 1).

Analysis of covariance (ANCOVA) showed that gender did not contribute significantly to the fit (Table 1).

**Table 1:** ANCOVA Analysis.

Coefficients	Estimate	Std. Error	t-value	p
**Intercept**	16.2508	0.6617	24.56	<0.001
**Gender**	-0.9197	0.9557	-0.962	0.344
**ROAR**	-75.671	5.6768	-13.33	<0.001
**Interaction**	9.663	7.634	1.266	0.216

Hence, including only ROAR in the regression model, the equation describing the known age of lions as a linear function yielded the following linear regression formula which explained 92.3% of total variance (R^2^ = 0.923):

Age  (in years)= −69.724 ROAR+15.781

**Fig 2:**
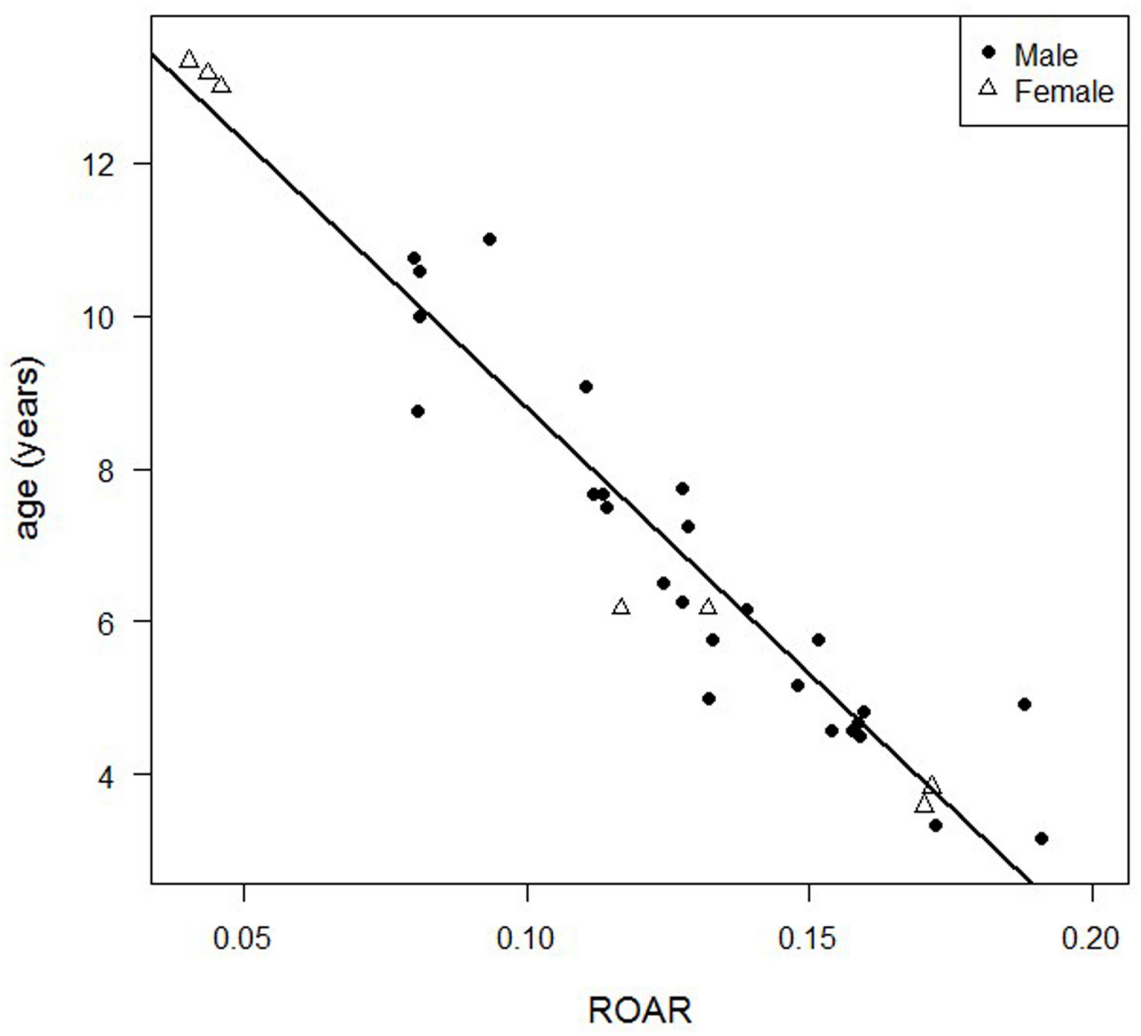
Relationship between Age and ROAR. Plot of the dataset used in the regression process to estimate age as function of ROAR, along with regression line.

**Fig 3:**
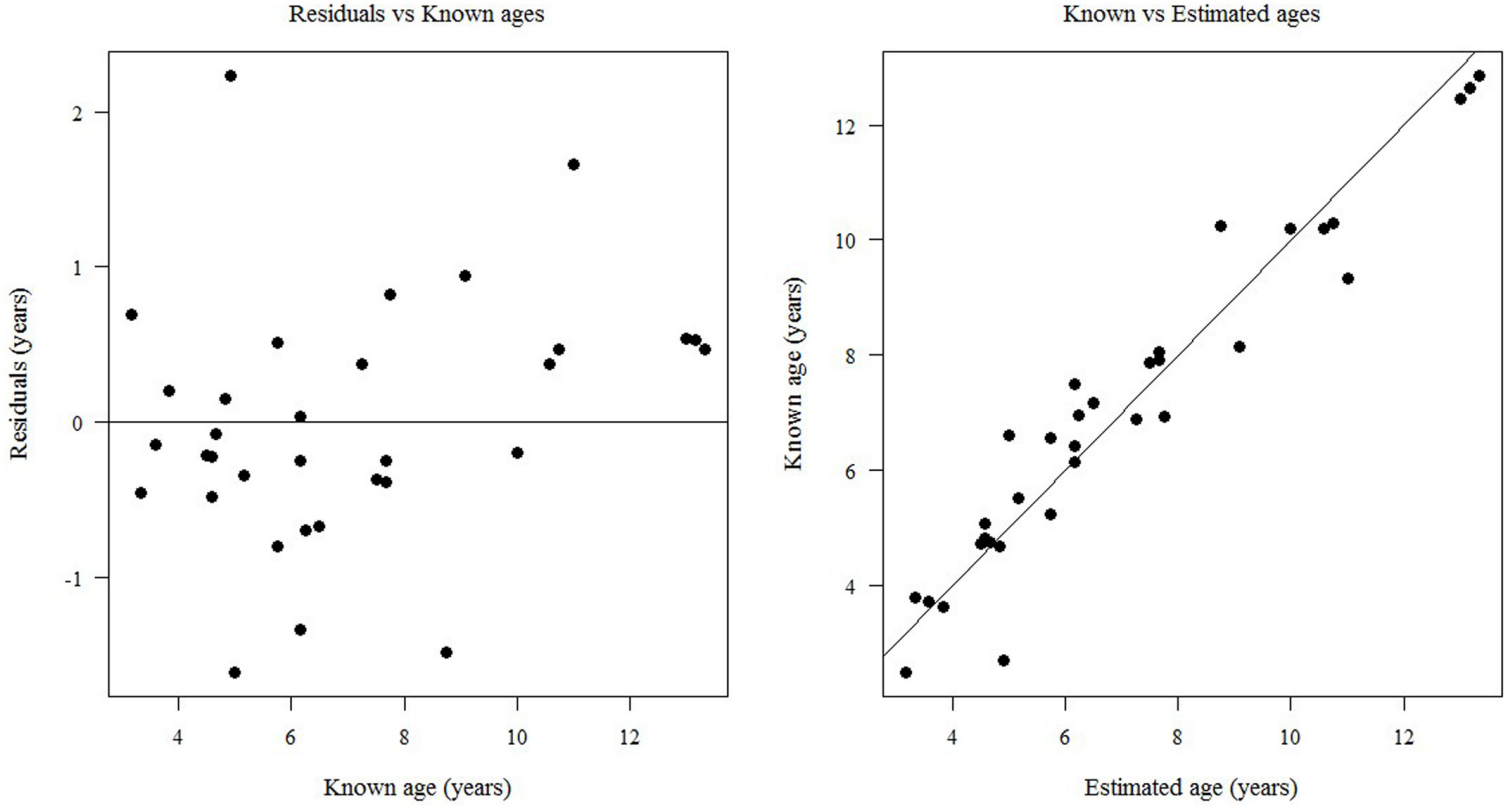
Plots of Residuals. Plots of residuals against known ages (left, a) and of known against estimated ages (right, b) using regression model.

The regression equation provided a point estimate of age for each given value of ROAR. The straight line obtained from reporting the estimated age as function of ROAR is presented in Fig 2. The residual standard error was 0.82 years and the median of the residuals was 0.43 years, with IQR = 0.38 years. The accuracy of the method was MAE = 0.60 years. The residual plot (Fig 3a) showed no obvious pattern, and only three observations appeared to be possible outliers. The plot of known versus estimated age (Fig 3b) showed that the regression model fits the trend of the data reasonably well. Hence, both diagnostic plots supported our chosen model.

Shapiro-Wilk test was not able to reject the hypothesis that residuals were independently drawn from a common normal distribution (p = 0.309). Grubbs’ test did not detect any outliers (p = 0.104). Finally, the value of R^2^_boot_ = 0.920, with its 95% bootstrapped confidence interval (CI = 0.843–0.969), pointed out the validity of our regression model.

## Discussion

Using known-aged animals, we have developed a linear regression formula for estimating age of African lions to within six months using ROAR of the PM^2^ as visualized from digital X-rays and quantified using area measurement software. To our knowledge, this is the first study to quantify pulp chamber closure of PM^2^s in known-aged lions. A previous attempt [28] to relate tooth and pulp area ratios of PM^2^s with age in lions (r^2^ = 0.46, 95% CI ± 2.5 years; combined sexes aged 1.3–10 years), used lions whose ages were derived from cementum line counts; a method that has recently been shown to be unreliable for estimating age in *P*. *leo* due to inconsistency of line counts between paired PM^2^s [29].

In contrast, ROAR appeared to work well in estimating ages of lions between the ages of 3–13 years old, with 92% of the total variance explained. Although one residual appeared greater than the others (Fig 3a), Grubbs’ test applied to the residuals did not indicate any outlier. Wild lions may occasionally live longer than 13 years, however by 10 years of age, most wild lions have severe wear or breakage of teeth [21, 22]. Crown damage that results in pulpal exposure can permanently halt dentine formation [21]. The linear regression formula provided a good fit through our dataset which included three (captive) lions 13 years of age. Thus, our model is applicable to lions up to the age of 13 years, perhaps older, provided that intact PM^2^s are available for X-ray analysis and measure. Smuts et al. (1978) reported that the pulp chamber in lion canines never becomes completely obliterated. Similarly, all three of the 13-year-old lions in this study retained a small opening of the pulp chamber visible on X-ray, suggesting that complete ossification of the PM^2^ may not occur in African lions, or not in all individuals. Alternatively, complete ossification of the PM^2^ may occur at an age older than 13 years.

Although sample size in this study was small, ROAR performed well on known-aged lions of both sexes from two regions (South Africa and Tanzania) living under different conditions (captive and wild, respectively). Closure of the apical foramina of canine teeth of female lions precedes that in males by a few months [21]. However in this study, an ANCOVA found no effect of gender on the chosen model. Also, although growth rate of captive lions is more rapid than that of wild lions due to nutrition [34], Smuts et al. (1978) found no difference in rates of pulp closure in the canine teeth of captive versus wild lions.

While Smuts et al.’s study provides the most comprehensive data to date on aging lions from teeth, several important differences exist between their study and the present one. Specifically, Smuts et al. (1978) examined pulp closure rates by measuring maximum pulp width in canines, on a dataset that included cubs. In contrast, our study measured total pulp/tooth areas in PM^2^s, and did not include lions under 3 years of age. Differences in morphology and developmental chronology between lion canine and PM^2^ teeth are vast. Thus, caution should be used when comparing characteristics of pulp chamber closure between studies.

While our findings suggest that ROAR may be broadly applicable to other lion populations, additional work is needed. In particular, continued testing of this regression formula to PM^2^s of known-aged lions from other populations and geographic regions is required to verify the accuracy of this method as a widespread tool. Increasing the sample size, especially in the 4–6-year-old age range that is of key importance to age-based trophy selection programs, and including lions older than 13 years would be prudent, and may allow further refinement of the model. Finally, development of Cameriere’s method to different tooth types e.g., canines and PM^3^s [32], may further increase the accuracy of lion age estimates.

Past studies conducted on humans have shown a strong correlation between pulp/tooth ratios and age [32, 35–37]. A recent study on wild African lion males found that pulp area measurements taken from below the crown to the root tip were similar (<4% variation) between an individual’s left and right PM^2^ pair [29]. Combined with the current study, these findings indicate that X-ray aging of either PM^2^ would provide consistent age estimates of lions. In this study, inter-observer repeatability for measuring X-rays was good and the error was small. While it is recommended that other morphological features related to age i.e., skull suture coalescence [21] be considered in post-mortem analyses, the refinement of lion age estimates to within six months on average using ROAR represents a substantial improvement over previously available techniques.

Development of regression formulas from pulp/tooth area ratios for age estimation appears to hold great promise and merits further exploration to determine if age assessments in other species can be improved through use of this method.

## Materials and Methods

### Data Collection

Tooth X-rays were obtained from cleaned lion skulls in South Africa (*n* = 25) and from live lions (*n* = 6) and skulls (*n* = 2) in Tanzania. South African lions were born in captivity and thus, their exact birth dates were known. Skulls of South African lions were examined and teeth X-rayed on-site at taxidermy studios. Age data on captive lions were obtained from the husbandry records kept by licensed animal-holding facilities in accordance with South Africa’s permitting requirements for Threatened and Protected Species (TOPS)[38] S2 Text.

In Tanzania, the ages of wild-living lions were known from long-term behavioural studies in Serengeti National Park [39] in which individuals were photographed at birth and thereafter identified and monitored over time using whisker spot patterns [40]. Skulls of known-age wild lions in Tanzania that died of natural causes and had been salvaged as part of the behavioural studies were examined and teeth X-rayed on-site in Serengeti.

The Tanzania Wildlife Research Institute (TAWIRI) is the national governing body that reviews and oversees all wildlife research in Tanzania. All proposed wildlife research activities must first obtain approval and official clearance by TAWIRI prior to commencement of field work. In addition to a description of the project goals and methods, research proposals must include number, sex, and age of animals involved in the project, as well as a justification of why the study is needed. The proposal to locate, dart, handle, and X-ray lions for this study was reviewed by TAWIRI’s Research Division and TAWIRI’s Veterinary Services Department, the latter of which includes review and consideration of animal care, use, and ethics. This study received full approval by TAWIRI for animal handling, care, use, and ethics prior to the initiation of field work.

Live lions in Tanzania were darted with anaesthetic and immobilized for X-raying using 300 mg of ketamine and 8 mg of medetomidine. The medetomidine was reversed using 40 mg of atipamezole and all lions fully recovered within a period of one hour. The chief TAWIRI veterinarian and a professional X-ray technician accompanied the Tanzanian field team and performed all veterinary procedures including darting, drugging, and monitoring of each lion throughout the handling and recovery period.

We chose second upper premolars (PM^2^s) for X-ray imaging as they are the tooth most commonly being used in African lion aging programs [19, 20, 29, 41]. Peri-apical X-rays of one PM^2^ from each of 33 lions (7 females, 26 males) aged 3–13 years old were analysed. Only single rooted PM^2^s without pathologies were chosen. Peri-apical digital X-rays were taken using a portable hand-held dental X-ray device (NOMAD Pro, Aribex Inc., East Orem, Utah) combined with a digital sensor (EVA Vet Size #2, ImageWorks, Elmsford, New York) linked to a portable laptop computer. All radiographs were taken with 0.05 s exposure time and 60 kV.

Following Cameriere et al. (2011) [33], the X-ray images were photo edited using Adobe Photoshop [42]. A step-by-step description of how to outline and calculate tooth and pulp areas using Photoshop, from Cameriere et al. 2011, is provided in S1 Text. Briefly, save the X-ray image as a high-resolution JPEG file and open in Photoshop. Using the polygonal lasso tool, click in the image at any point along the outer edge of the PM^2^ to set the starting point for measuring the tooth area. Move the cursor to a close point along the tooth profile, and click again. A straight line from the first point selected will be drawn. Continue clicking to set endpoints for subsequent segments, placing a point at each change of direction and angle of the tooth, until the entire tooth has been outlined. Note that there are no specific locations at which points must be selected; the placement of points is dictated by the shape and angles that vary with each individual tooth. The last segment connects to the starting point to enclose the tooth area measure. Repeat using a new starting point to obtain a separate outline for the pulp chamber area measure, demarcating the outline of the dark inner portion of the root that corresponds to the open pulp chamber (Fig 1). A minimum of 20 points should be selected to delineate the PM^2^ area, and a minimum of 10 points to delineate the pulp chamber area. Save the tooth and pulp area outlines as separate layers, and use the histogram palette of Photoshop to read the number of pixels contained in each layer. The number of pixels in the two layers represents the two values from which the pulp/tooth area ratio is calculated.

### Statistical analysis

For each lion, the known age was calculated by subtracting date of birth from the date of X-ray for live lions, or date of death for lion skulls.

Dental maturity was evaluated by measuring the extent of pulp closure as a ratio of tooth area (ROAR) of the PM^2^. All measurements were carried out by the same observer. To test intra-observer reproducibility (ICC), a random sample of 15 peri-apical X-rays was re-examined after an interval of two weeks. The ICC, and its non-parametric bootstrapped 95% confidence interval (95% CI), was used to evaluate intra-observer agreement. The intervals were based on 1000 bootstrap replicates.

To analyze the relationship between known age and dental maturity, we performed a linear regression analysis with known age as dependent variable and ROAR as independent variable. Furthermore, to evaluate the accuracy of the regression model, for each lion the known age (*Age*_*i*_, *i = 1*,*…*,*n*; with *n* = 33) was compared to the estimated age (*Age*_*est*,*i*_, *i = 1*,*…*,*n*; with *n* = 33) using the mean absolute prediction error:

$$MAE=\frac{1}{n}\sum_{i=1}^{n}\left|Age_{i}-Age_{est,i}\right|$$

Analysis of covariance (ANCOVA) was then applied to study possible interactions between significant variables and gender.

To measure model performance, taking into account the small sample size, we did not divide the data into separate training and test sets. Hence, all the estimates were obtained using all the available data, and the performance of the model was evaluated by a bootstrap resampling strategy [43]. We used the bootstrap for validation purposes by estimating the optimism of R^2^ as an index of predictive accuracy. Optimism is a positive bias in predictive accuracy, which usually occurs when we assess predictive accuracy on the same data set used to fit the model. Lastly, subtracting the estimated expected optimism from the model R^2^, we obtain bootstrap R^2^, R^2^_boot_, which estimates the predictive accuracy of the model.

Statistical analyses were performed with the statistical program R Core Team [44]. The significance threshold was set at 5%.

## Supporting Information

S1 Text Step-by-step instructions on how to use Adobe Photoshop to obtain measures of tooth and pulp areas, after Cameriere et al. 2011. (DOCX)

S2 Text Lion breeding facility and taxidermy studios. (DOCX)
