# Correction: Age Estimation of African Lions *Panthera leo* by Ratio of Tooth Areas

**DOI:** 10.1371/journal.pone.0187003

**Published:** 2017-10-19

**Authors:** Paula A. White, Dennis Ikanda, Luigi Ferrante, Philippe Chardonnet, Pascal Mesochina, Roberto Cameriere

In assessing the relationship between African lions of known age and the extent of pulp closure in the PM^2^ as viewed from X-rays, the authors earlier reported a formula derived from a linear regression of the Ratio Of tooth AReas (ROAR) and lion age in years [1]. Subsequently, the original formula was found to contain an error in the tooth/pulp area ratios. Herein, the authors present a corrected formula and revised results, based on the same dataset of known aged lions aged 3–13 years old re-measured using the Adobe Photoshop magnetic lasso tool that greatly reduces subjectivity of outlining the tooth and pulp areas.

The re-calculated intra-class correlation coefficients (ICC) of 0.999 (95%CI: 0.998–1.000) for tooth areas and 0.998 (95% CI: 0.996–0.999) for pulp areas measured using the magnetic lasso tool indicated a high level of intra-observer consistency among measurements carried out on re-examined X-rays.

Overall, the authors found a significant relationship between known lion age and the corrected ROAR (ANCOVA; F_2,30_ = 69.824, p < 0.001), and the effect of gender was not significant (F_1,30_ = 0.373, p = 0.546).

Hence, including only ROAR in a simple linear regression model, the equation describing the known age of lions as a linear function yielded the following formula which in the corrected equation explains 51.0% of total variance (R^2^ = 0.5096):

Age (years)= −72.559 ROAR+14.814

The image for Fig 2 has been corrected accordingly. Please see the correct Fig 2 here.

**Fig 2:**
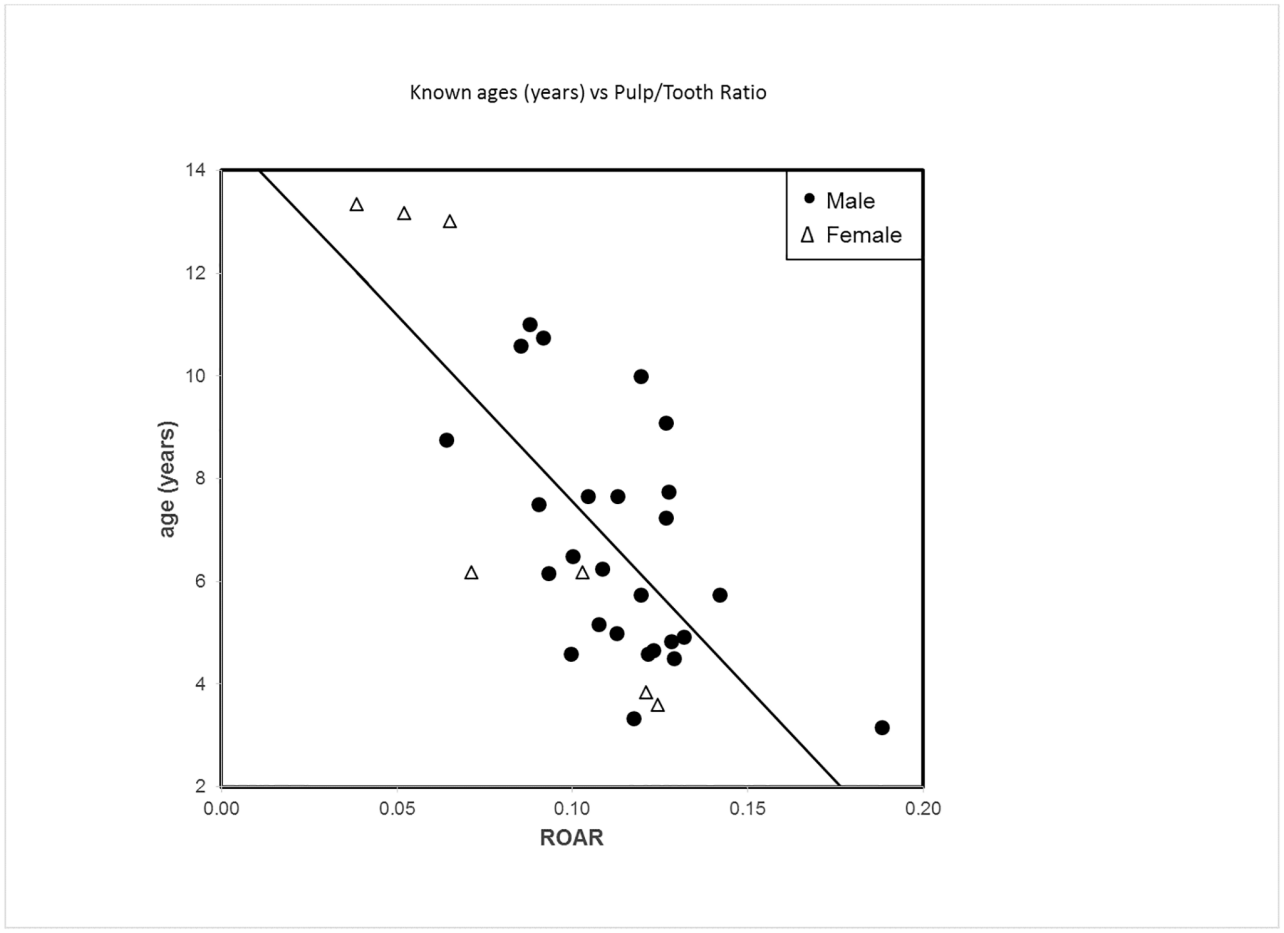
Relationship between age and ROAR. Plot of the dataset used in the regression process to estimate age as a function of ROAR, along with regression line.

The residual standard error was 0.16 years and the median of the residuals was 1.66 years, with IQR = 3.49 years. The accuracy of the method was MAE = 1.78 years. A Shapiro-Wilk test suggested the residuals were normally distributed (W = 0.951, p = 0.138). Grubbs’ test did not detect any outliers (p > 0.050).

The image for Fig 3 has been corrected accordingly. Please see the correct Fig 3 here.

**Fig 3:**
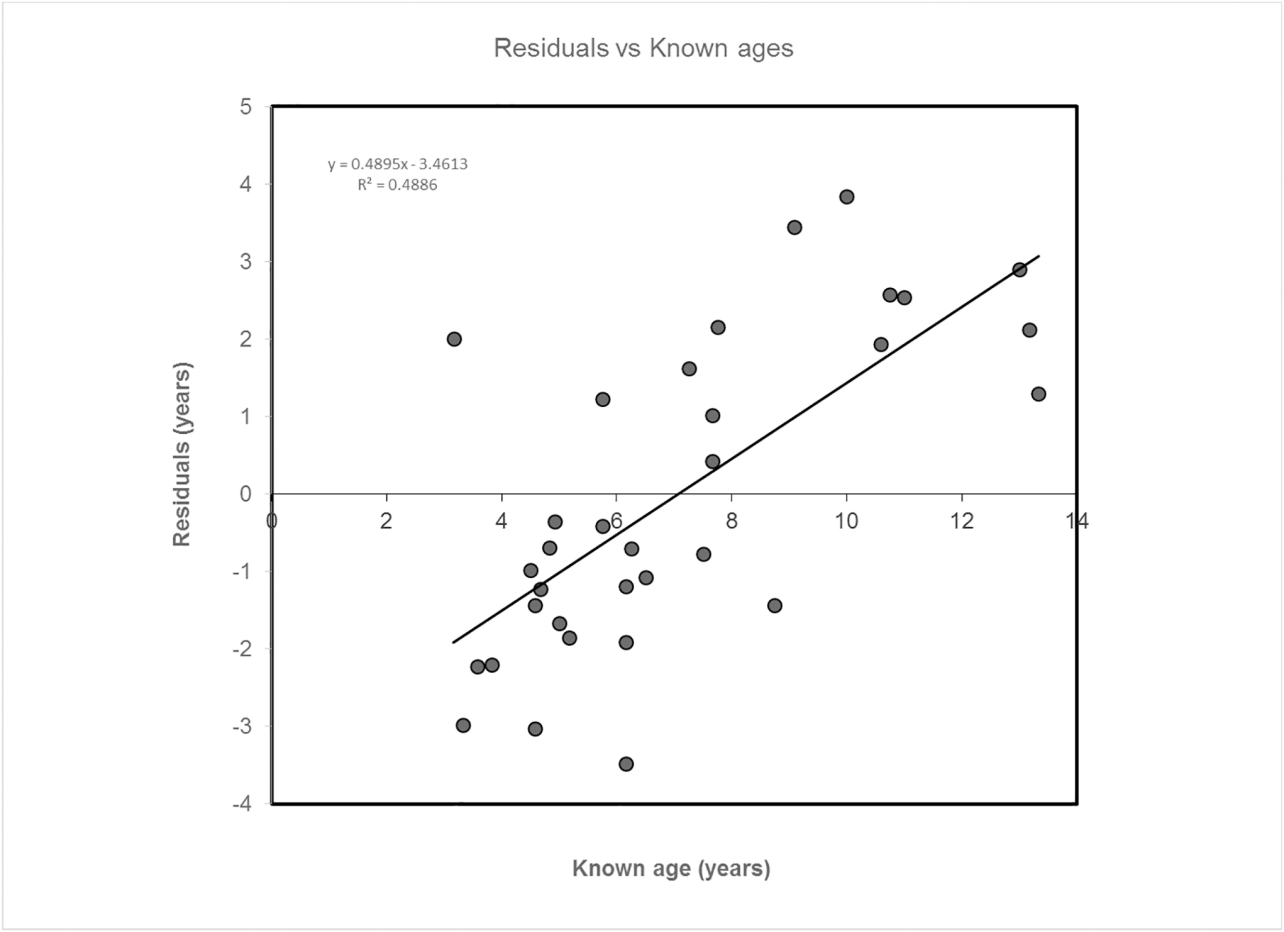
Plots of residuals. Plots of residuals against known ages using regression model.

Given the reduced fit of the corrected regression, the authors tested ROAR’s accuracy in assigning individual lions to age classes. ROAR correctly assigned 22 of 23 (96%) lions aged ≥ 5 years to the “above 5 years” age class (positive predictive value (PPV) = 0.71), although it did not distinguish into which older age class the lion belonged. However, lions aged < 5 years were consistently (9 of 10) assigned incorrectly to older ages classes (negative predictive value (NPV) = 0.50). This included three out of four lions aged < 4 years. The authors have provided a new figure for these data. Please see Fig 4 here.

**Fig 4:**
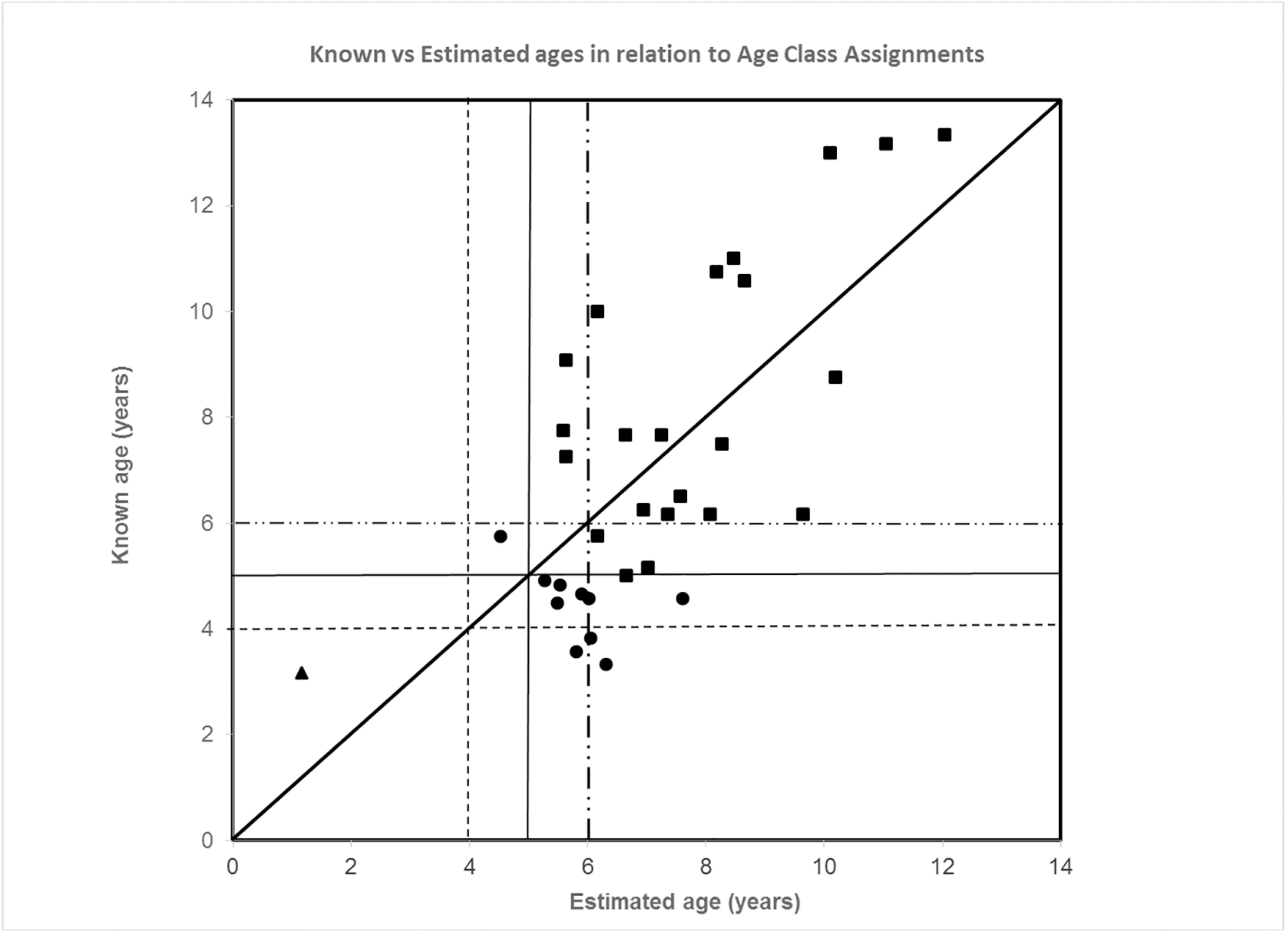
Accuracy of ROAR in assigning individuals to age class. Plot of estimated age versus known age as it relates to age class. Dotted vertical and horizontal lines demarcate the 4-year-old age class; solid lines demarcate the 5-year-old age class; dashed lines demarcate the 6-year-old age class. The solid diagonal line through the origin denotes an age estimate that corresponds exactly to the known age. Triangles indicate lions assigned correctly to the < 5 year age class, squares indicate lions assigned correctly to the ≥ 5 year age class, and circles indicate lions assigned to an incorrect age class.

ROAR offers a quantifiable measure of the relationship between pulp/tooth area ratios of the PM^2^ and lion age, yet the standard error precludes assignment of individual lions to specific age in years and, in some cases, to class. Although the margin of error is similar to that encountered in human aging studies [2–5], the comparatively shorter life span of African lions renders the margin of error proportionately greater. In particular, incorrect assignment of lions less than five years of age to older age classes is detrimental to management programs seeking to ensure that offtakes are sustainable [6, 7]. Therefore, while further investigation into the above relationship is warranted, it is recommended that for African lions the use of pulp chamber closure of the PM^2^ for age assessment be considered in conjunction with other age-related characteristics [8, 9] until further refinements can be made.
