# Supplementary material for: Age Estimation of African Lions Panthera leo by Ratio of Tooth Areas
Source: PLoS One. 2016 Apr 18;11(4):e0153648. doi: 10.1371/journal.pone.0153648 (PMC4835051; doi:10.1371/journal.pone.0153648)
Supplement: S1 Text — (DOCX) [file pone.0153648.s001.docx]

S1 Text. Supporting Information: Save the radiographic images as high-resolution JPEG files on a desktop computer and import to Adobe Photoshop CS41 image-editing software program (Adobe Systems Incorporated, San Jose, CA, USA). Next, open the image file, enlarge the working area, and zoom in. Adjust brightness/contrast and sharpness if needed. Some X-ray images are sometimes over- or underexposed or a little blurred. With the aid of some Photoshop tools, it is possible to correct some image problems or enhance some edges not clearly visible. Select the polygonal lasso tool from the tool bar. In order to select the entire premolar area, click in the premolar image to set the starting point of the premolar shape; move the cursor to a close point of the tooth profile and click again. A straight line from the first point selected will be drawn. Note that there are no specific locations at which points must be selected; the placement of points is dictated by the shape and angles that varies with each individual tooth. Continue clicking to set endpoints for subsequent segments along the premolar profile. A minimum of 20 points from each tooth outline has now been identified and connected with the line tool. Now copy and paste the selected area on a new layer, which will be added to the active working area superimposed on the premolar image. This new layer, renamed ‘‘PREMOLAR’’, will be added to the layer palette. In order to select the pulp chamber area, proceed as previously for the entire premolar, following the pulp chamber profile with the polygonal lasso. A minimum of 10 points will also have been marked on the pulp outline, although up to 15 points are marked in some cases. Copy and paste the pulp chamber selection to a new layer and rename it ‘‘PULP CHAMBER’’. The new ‘‘PULP CHAMBER’’ layer contains only the premolar pulp chamber area pixels, as the ‘‘PREMOLAR’’ layer contains the entire premolar area pixels. To know how many pixels there are in each layer, activate the histogram palette (windows > histogram) and the ‘‘PULP CHAMBER’’ layer by clicking on the layer name in the layer palette. In the histogram palette from the source menu, now choose SELECTED LAYER and double-click on the histogram image. Read the number of pixels contained in the ‘‘PULP CHAMBER’’ layer in the histogram palette. This value represents the first needed variable (pulp chamber area). Next, select the ‘‘PREMOLAR’’ layer, double-click on the histogram image and read the number of pixels contained in the entire premolar. This value represents the second needed variable.
