# Supplementary material for: Age Estimation of African Lions Panthera leo by Ratio of Tooth Areas
Source: PLoS One. 2016 Apr 18;11(4):e0153648. doi: 10.1371/journal.pone.0153648 (PMC4835051; doi:10.1371/journal.pone.0153648)
Supplement: S2 Text — (DOCX) [file pone.0153648.s002.docx]

S2 Text. Supporting Information: Skulls of South African lions aged in this study were X-rayed at Moredou Taxidermy, Schweizer Reneke, South Africa and LifeForm Taxidermy, White River, South Africa where they were being cleaned and prepared as components of private hunting trophies. Permissions to gain access and to age lion skulls at each location were granted by the owners of the taxidermy studios. Upon completion of taxidermy preparation, individual skulls were delivered to clients as private hunting trophies and thus, are no longer available for inspection; however, X-rays of all lion teeth used in this study are available from the corresponding author (PAW) upon request. South African lions aged in this study were born in captivity at Thandeka Safaris property, a government licensed predator breeding facility and hunting ranch in Tosca, North West Province, South Africa, S 25^o^ 42min; E 023^o^ 58min. (www.thandekasafaris.co.za). The 25 South African lion skulls aged in this study originated from trophy hunted lions that were transported to taxidermists for trophy preparation following the hunts. Permissions to age lion skulls at the taxidermy studios, and age data records for this study, were provided by the owner and facility manager of Thandeka Safaris, respectively.
